# Tip-end fusion of a rod-shaped secretory organelle

**DOI:** 10.1007/s00018-022-04367-2

**Published:** 2022-06-04

**Authors:** Johannes Naß, Sophia N. Koerdt, Anja Biesemann, Tarek Chehab, Takao Yasuda, Mitsunori Fukuda, Fernando Martín-Belmonte, Volker Gerke

**Affiliations:** 1 Institute of Medical Biochemistry, Center for Molecular Biology of Inflammation, University of Muenster, von-Esmarch-Str. 56, 48149 Muenster, Germany; 2 Department of Integrative Life Sciences, Graduate School of Life Sciences, Tohoku University, Sendai, Miyagi 980-8578 Japan; 3 Centro de Biologia Molecular Severo Ochoa, Spanish National Research Council (CSIC), Lab 427, C/Nicolas Cabrera 1, 28049 Madrid, Spain

**Keywords:** Calcium, Endothelial cells, Exocytosis, Von-Willebrand factor, Weibel–Palade bodies

## Abstract

Weibel–Palade bodies (WPB) are elongated, rod-like secretory organelles unique to endothelial cells that store the pro-coagulant von-Willebrand factor (VWF) and undergo regulated exocytosis upon stimulation with Ca^2+^- or cAMP-raising agonists. We show here that WPB preferentially initiate fusion with the plasma membrane at their tips and identify synaptotagmin-like protein 2-a (Slp2-a) as a positive regulator of VWF secretion most likely mediating this topological selectivity. Following secretagogue stimulation, Slp2-a accumulates at one WPB tip before fusion occurs at this site. Depletion of Slp2-a reduces Ca^2+^-dependent secretion of highly multimeric VWF and interferes with the formation of actin rings at WPB–plasma membrane fusion sites that support the expulsion of the VWF multimers and most likely require a tip-end fusion topology. Phosphatidylinositol (4,5)-bisphosphate [PI(4,5)P_2_] binding via the C2A domain of Slp2-a is required for accumulation of Slp2-a at the tip ends of fusing WPB, suggesting that Slp2-a mediates polar exocytosis by initiating contacts between WPB tips and plasma membrane PI(4,5)P_2_.

**Supplementary Information:**

The online version contains supplementary material available at 10.1007/s00018-022-04367-2.

## Introduction

Weibel–Palade bodies (WPB) are specialized lysosome-related secretory organelles of endothelial cells. They store and secrete the pro-coagulant glycoprotein von-Willebrand factor (VWF), which is the defining cargo of WPB and whose highly multimeric tubular assembly determines the characteristic rod-like shape of WPB. Upon exocytosis, VWF unfolds into VWF strings that provide a scaffold for platelets to efficiently form a platelet plug inside injured blood vessels (for reviews, see [1–4]). P-selectin and CD63, which are transmembrane proteins of WPB [5, 6], are presented on the endothelial cell surface upon WPB exocytosis, and then support interactions between leukocytes and the vessel wall [7, 8]. Therefore, WPB exocytosis, which is triggered by secretagogues elevating intracellular Ca^2+^ or cAMP, rapidly transforms the normally anti-adhesive surface of blood vessels into an adhesive one capable of capturing leukocytes and platelets (for a review, see [9]). Hence, acute WPB exocytosis must be tightly controlled and activated only at times of injury to blood vessels, in inflammatory conditions, or upon other types of insult.

WPB form at the trans-Golgi network and undergo a maturation process that involves a tight packing of VWF tubules resulting in the rod-like morphology. Maturation is also accompanied by the recruitment of Rab GTPases including Rab3 isoforms and in particular Rab27a, a long-known marker for WPB [10–15]. These, in turn, recruit effector proteins, which regulate WPB dynamics and exocytosis. One Rab27a effector implicated in WPB maturation is Myosin VIIA And Rab Interacting Protein (MyRIP), also known as synaptotagmin-like protein lacking C2 domains-c (Slac2-c), which anchors WPB at the cortical actin cytoskeleton, thereby preventing the exocytosis of premature WPB [16–18]. Synaptotagmin-like protein 4-a (Slp4-a), an effector of Rab3 and Rab27 isoforms, promotes WPB exocytosis following secretagogue stimulation [10]. It functions by interacting with syntaxin-binding protein 1 (STXBP1), also known as Munc18-1, which in turn binds SNARE proteins including syntaxin 3 [19]. The involvement of SNARE complexes in WPB exocytosis is illustrated by the dependency of VWF secretion on *N*-ethylmaleimide-sensitive factor (NSF) [20–22]. Candidates for the catalytic SNARE complex mediating WPB exocytosis include, in addition to syntaxin 3, VAMP3, VAMP8, syntaxin 4, and SNAP23 [19, 21, 23–25]. Plasma membrane (PM) lipids, specifically phosphatidic acid (PA) and phosphatidylinositol(4,5)-bisphosphate (PI(4,5)P_2_), also play an important role in mediating the WPB fusion. Both accumulate at sites of WPB exocytosis and enzymes generating these lipids, phospholipase D1 (PLD1) and PI4P 5-kinase (PI4P5Kγ), positively support WPB exocytosis and VWF secretion [26, 27]

Different modes of WPB–PM fusion have been described. They include full fusions where the WPB membrane flattens completely into the PM and all cargo is released, and kiss-and-run fusion events that permit transient fusion pore opening and selective release of (smaller) WPB cargo. Other modes of WPB exocytosis are compound fusions where individual WPB fuse inside the cell to form a secretory pod that eventually fuses with the PM, cumulative/sequential fusions characterized by a second WPB fusing to a first that had already fused with the PM, and cavicapture-like events that are characterized by fusion pores allowing the release of large cargo like VWF but restricting the WPB membrane from fully collapsing into the PM [3, 28–30]. Interestingly, at least the latter events are accompanied by an actin ring or coat that forms at the base (PM-distal) of the fused WPB membrane and supports the release of the large VWF cargo through actomyosin contractility [31].

Given the unique, rod-like shape of WPB and previous cryo-EM studies that suggested a tip-end orientation of peripheral WPB [32], we addressed the question of whether WPB prefer a specific topology when fusing with the PM, i.e., whether they fuse randomly, or preferentially with either the tip or the lateral side pointing toward the membrane. We show that tip-end fusion is highly favored and consider this topology relevant as it could affect the release of small versus large WPB cargo and the assembly of actin rings at the base of fused WPB. In search of factors that confer such topical selectivity, we identify synaptotagmin-like protein 2-a (Slp2-a), a Rab27a effector that recently has been linked to WPB exocytosis [33]. Slp2-a serves as a positive regulator of the secretion of highly multimeric VWF and upon secretagogue stimulation becomes concentrated at the WPB tip which will undergo fusion. Slp2-a is known to bind phosphatidylserine (PS) and PI(4,5)P_2_ [34] and we show that PI(4,5)P_2_ binding is required for tip-end localization of Slp2-a at fusing WPB. The Slp2-a mediated tip-end fusion topology is important for an assembly of actin rings at the base of fused WPB that supports the expulsion of highly multimeric forms of VWF as both events are compromised in endothelial cells depleted of Slp2-a.

## Results

### Topology of WPB fusion

**Fig. 1:**
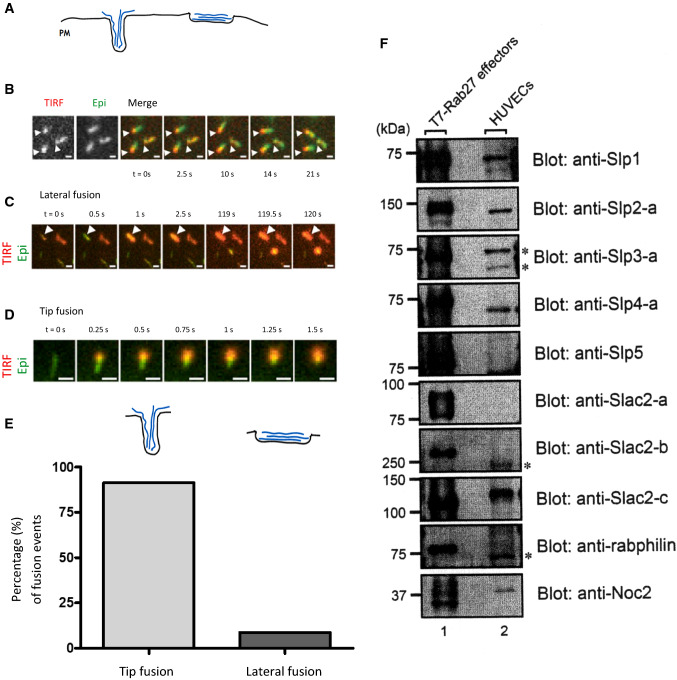
WPB preferentially undergo tip fusion. **A** Scheme depicting the extreme scenarios of WPB tip fusion (left) and lateral fusion (right). black: membrane; blue: VWF. **B–D** HUVEC transfected with VWF-EGFP were imaged with simultaneous TIRF (red) and epifluorescence (epi, green) illumination, and stimulated with 20 µM histamine during acquisition. Scale bars = 1 µm. **B** WPB can appear fixed with one end within the TIRF field (arrowheads) whereas the rest of the WPB moves freely. **C** WPB aligned along its length toward the plasma membrane and producing an elongated fusion spot (lateral fusion: arrowhead; see also Video 1). **D** WPB fusing with one end first and collapsing into a round fusion spot (tip fusion; see also Video 2). **E** Frequency of any kind of tip fusion as compared to extreme events of full lateral fusion of *n* = 267 fusion events in seven cells (9 out of 267 were considered lateral fusion, i.e., 3.4%). Fusions were only counted as lateral fusions when WPB were clearly aligned along their length before fusion and the resulting fusion spots were at least twice as long as wide. **F** HUVEC total lysates probed with antibodies against the proteins indicated, with T7-tagged, overexpressed proteins as positive controls, which ensures that the antibodies recognize the target proteins. Several bands of unexpected sizes were observed in the Slp3-a, Slac2-b, and rabphilin blots (indicated by asterisks). These bands presumably correspond to non-specific signals. However, we cannot completely rule out the possibility that these bands represent unreported splice variants of Rab27 effectors or their degradation products

The fusion of a rod-shaped WPB with the plasma membrane (PM) can, in principle, proceed via two morphologically distinct events: the fusion pore could open either on the lateral organelle surface, or on one of the organelle’s approximately hemispherical tips (Fig. 1A). To quantify the relative numbers of lateral and tip WPB fusions in primary human umbilical vein endothelial cells (HUVEC), we performed time-lapse live-cell microscopy with simultaneous epifluorescence and TIRF illumination of VWF-EGFP marked WPB stimulated for exocytosis with histamine during acquisition. In these conditions, WPB often appear tethered at or very close to the PM even before fusion. In such cases, one of the tips of the WPB is visible in both the TIRF and epifluorescence channels, while the rest of the organelle is only visualized with epifluorescence illumination, indicating that it extends away from the evanescent TIRF illumination field and into the cell interior (Fig. 1B). Over time, the position of the WPB tip that is closest to the PM remains fixed, even when the “tail” of the organelle rotates around the “head” portion of the organelle within the TIRF field (Fig. 1B).

At an acquisition rate of 4 frames per second, we were able to identify two distinct categories of fusion events. Some fusion events were consistent with a lateral fusion mode as they presented an oblong fusion spot that essentially preserved the orientations of the long and short axes of the pre-fusion WPB (Fig. 1C, Video 1). These WPB were mostly located within the TIRF field across their entire length and thus oriented parallel to the coverslip. On the other hand, we observed fusion events where the bright fusion spot remained confined to a roundish area at the place where the WPB had been within the TIRF field before fusion, consistent with tip fusions (Fig. 1D, Video 2). In the latter case, the part of the organelle that was distal to the fusion pore often retained its elongated shape for a few seconds before collapsing into the fusion spot. Of note, we did not observe WPB that had round fusion spots in their middle with non-collapsed elongated parts of the WPB extending to either side still visible after fusion. We quantified the relative frequencies of types of fusion events and found that overall, less than 5% of WPB fusions are unambiguously lateral, whereas the other fusion events showed aspects of a round fusion spot (Fig. 1E). Together, these data show that WPB preferentially fuse with the PM in a “head on” orientation. They furthermore imply that one WPB tip often becomes tethered to a PM proximal site before fusion.

### Slp2-a: an Rab27a effector localizing to the fusion-competent tip of WPB in stimulated HUVEC

**Fig. 2:**
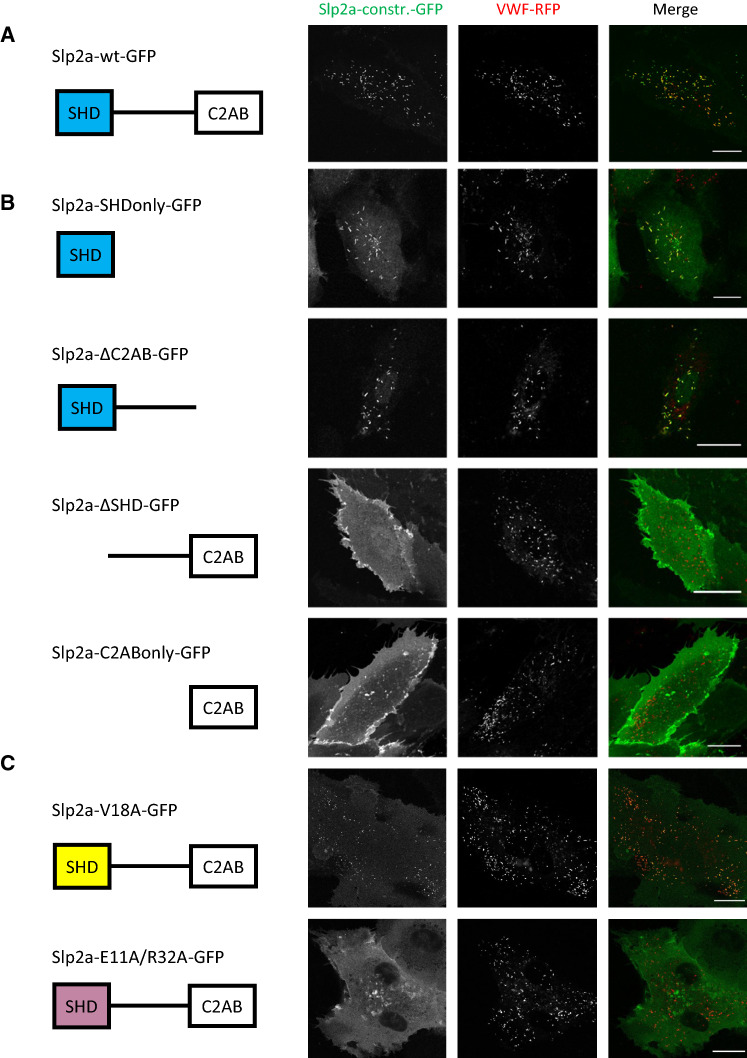
Slp2-a localizes to WPB by binding Rab27 via the SHD. **A–C** HUVEC were transfected with the indicated Slp2a-GFP constructs and VWF-RFP. Shown are stills of the respective live-cell recordings. Scale bars = 20 µm

Next, we searched for proteins that might confer fusogenic functionality to the WPB tip. As Rab27a is a protein specifically recruited to mature elongated WPB residing in the cell periphery [12], we concentrated on Rab27a effectors as candidates for mediating a tip-end fusion. We, therefore, assessed the presence of a panel of Rab27a effectors in HUVEC lysates by Western blot. We confirmed the expression of MyRIP and Slp4-a [10, 31], and furthermore identified the presence of Slp1, Slp2-a, and Noc2 at the protein level (Fig. 1F). Among these, Slp2-a is known to play a role in targeting secretory granules to the PM of pancreatic α cells [34] and gastric-surface mucous cells [35], affecting granule secretion in cytotoxic T lymphocytes (CTLs) [36, 37] and, as recently shown, regulating the exocytic activity of WPB [33]. In addition to binding Rab27a via its N-terminal SHD (Slp homology domain), Slp2-a also binds Ca^2+^ and interacts with PS and PI(4,5)P_2_ via its C-terminal C2 domains [34] and thus could potentially function as a Ca^2+^-regulated bridge between WPB and the PS/PI(4,5)P_2_-rich PM. Therefore, we first verified that Slp2-a associates with WPB in HUVEC by expressing Slp2-a fused to GFP together with VWF-RFP. Slp2a-wt-GFP clearly co-localized with the VWF-RFP signal on WPB (Fig. 2A). We did not observe any obvious changes in WPB number, localization, or morphology upon Slp2a-GFP expression. Next, we analyzed the subcellular localization of several Slp2-a truncation mutants (overview of the constructs used in Fig. 2). While the Slp2a-SHDonly-GFP and Slp2a-ΔC2AB-GFP mutants co-localized with WPB in a manner similar to the wild-type construct, Slp2a-ΔSHD-GFP and Slp2a-C2ABonly-GFP were found mainly on the PM and showed no WPB staining (Fig. 2B). These data confirm that the Rab27a interacting SHD is necessary for recruiting Slp2-a to WPB, while the C2 domains are recruited to the plasma membrane, mediated possibly by PI(4,5)P_2_ and/or PS binding of the Slp2-a C2A domain [33].

To further analyze the role of Rab27a in recruiting Slp2-a to WPB, we used point mutants within the SHD of Slp2-a known to disrupt either binding to Rab27, Rab3, and Rab8 (Slp2a-E11A/R32A) or selectively to Rab3 and Rab8 only (Slp2a-V18A) [38]. While Slp2a-E11A/R32A-GFP assumed cytosolic and plasma membrane localization, Slp2a-V18A-GFP localized to WPB, with some general plasma membrane/cytosolic distribution (Fig. 2C). Moreover, depletion of Rab27a in HUVEC resulted in a loss of WPB association of Slp2a-wt-GFP (Fig. S1). These data demonstrate that recruitment of Slp2a-GFP to WPB depends on Rab27a, but not on Rab8 or Rab3 isoforms.

**Fig. 3:**
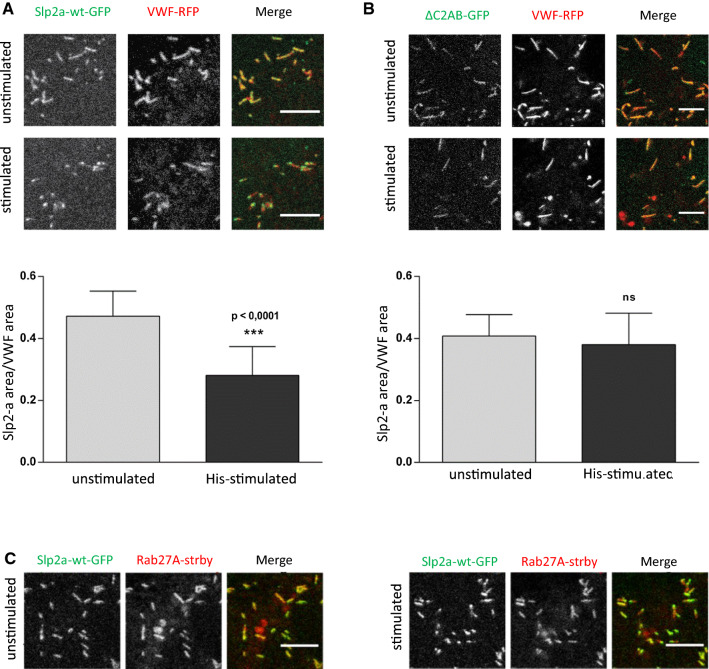
Slp2 assumes tip localization after histamine stimulation independent of Rab27a. **A**, **B** HUVEC transfected with Slp2a-wt-GFP or Slp2a-ΔC2AB-GFP and VWF-RFP were imaged by confocal time-lapse microscopy and stimulated with 100 µM histamine during acquisition (see also Video 3 and 4). Stills of the stimulated condition represent a time-point after a few WPB fusions had already occurred. Tip localization was quantified as described in “Materials and methods” section from *n* ≥ 11 cells; error bars = SD. Significance was tested with paired Student’s *t* test (****p ≤ 0.0001, ns = not significant). **C** HUVEC transfected with Slp2a-wt-GFP and Rab27a-strby were imaged by confocal time-lapse microscopy and stimulated with 100 µM histamine during acquisition. The stimulated condition represents a time-point after a few WPB fusions had already occurred. Scale bars = 5 µm

To assess the dynamics of Slp2-a localization during WPB fusion, we performed time-lapse live-cell confocal microscopy of HUVEC transfected with Slp2a-GFP and VWF-RFP. In this setting, individual WPB–PM fusion events can be visualized by the collapse of the elongated VWF-RFP signal into a round fusion spot [29, 39]. Upon addition of histamine, Slp2a-GFP accumulated on most WPB in distinct puncta, with one Slp2a-GFP puncta per WPB that were usually located at one of the tips of the organelle (Fig. 3A, Video 3). We will refer to this phenomenon as Slp2-a “tip localization” in the following.

To study the role of the C2 domains in Slp2-a tip localization, we performed live-cell confocal microscopy with Slp2a-ΔC2AB-GFP. In contrast to wild-type Slp2-a, we did not observe any evidence of Slp2a-ΔC2AB-GFP tip localization (Fig. 3B, Video 4). We quantified tip localization of the wild-type and ΔC2AB constructs by assessing the degree of overlap between their respective fluorescent signals and that of VWF-RFP in resting cells, and again shortly after stimulation but before many WPB had fused. The degree of co-registration of Slp2-a and VWF positive pixels is significantly reduced after stimulation for the wild type, indicative of tip localization (Fig. 3A). In contrast, co-registration remains virtually the same for the ΔC2AB construct before and after histamine stimulation (Fig. 3B).

**Fig. 4:**
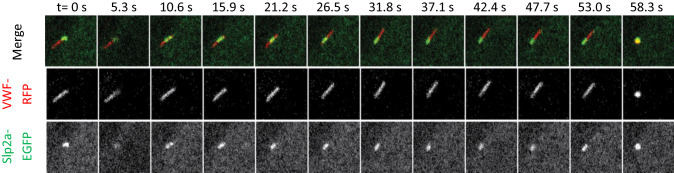
The Slp2-a-positive tip of WPB is the site of fusion initiation. HUVEC transfected with Slp2a-wt-GFP and VWF-RFP were imaged by confocal time-lapse microscopy and stimulated with 400 µM histamine during acquisition (see also Video 5). Stills shown correspond to the indicated time-points after histamine stimulation. At *t* = 0 s, Slp2a has assumed tip localization; the Slp2-a accumulation then moves to the opposing tip (frames 5.3–31.8 s); finally, WPB fusion occurs at the Slp2-a-positive tip (frame 58.3 s). The ‘tip end flipping’ shown here is a rare event and most likely occurs because the initial accumulation of Slp2-a at one tip is not firmly established at this point. Picture size is 7.3 × 7.3 µm

We next assessed whether Slp2-a tip localization is secondary to a potential tip localization of its binding partner Rab27a. Therefore, we co-transfected HUVEC with Rab27a-strby and Slp2a-GFP and again performed live-cell microscopy. After histamine stimulation, Slp2a-GFP clearly assumed tip-localization, whereas the Rab27a-strby signal remained in the typical WPB shape until fusion (Fig. 3C). Collectively, these data suggest that Slp2-a tip localization on WPB is triggered by histamine stimulation, independent of Rab27a, and rather mediated by the Slp2-a C2AB domains. A careful observation of the individual WPB fusion events revealed that the WPB tip positive for Slp2a-GFP was the part of the organelle that fused with the plasma membrane (Fig. 4, Video 5). We, therefore, hypothesized that Slp2-a, via the lipid-binding capability of its C2 domains, establishes contact with the plasma membrane after Ca^2+^-elevation, and thereby confers special fusion competence to the WPB tip it accumulates on.

Recently, Sharda et al. [40] reported that the exocyst complex, in addition to supporting the trafficking of endolysosomal components to maturing WPB which is mediated by interactions with the biogenesis of lysosome-related organelle-2 (BLOC-2), can act at the plasma membrane as a clamp impeding WPB exocytosis. As this exocyst action could benefit from a tip-end orientation of WPB [40], we also analyzed a potential involvement of exocyst in Slp2-a tip-end enrichment. However, exocyst inhibition by the EXO70-binding Endosidin2 had no significant effect on the histamine-induced enrichment of Slp2-a at WPB tips (Fig. S2).

### Slp2-a is a positive regulator of the secretion of highly multimeric VWF

**Fig. 5:**
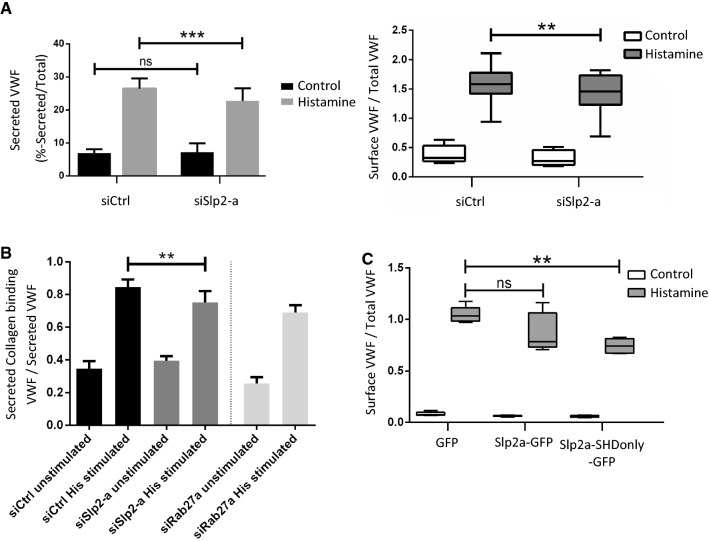
Slp2 is a positive regulator of VWF secretion. **A** Left: Quantification of VWF secretion efficiency after Slp2-a depletion. HUVEC were transfected with 400 pmol siCtrl or siSlp2 for 48 h, and again with the same amount of the respective siRNA. 48 h after the second transfection, cells were treated with agonist-free medium and then histamine-containing medium for 20 min each. After cell lysis, VWF contained in the agonist-free and histamine-stimulated cell culture supernatant as well as in the cell lysate, respectively, was quantified by ELISA. VWF secretion was normalized to total cellular VWF content. *n* = 8 experiments, significance tested with paired Student’s *t* test (***p ≤ 0.001). Right: Quantification of VWF secretion via an anti-VWF antibody capture assay. HUVEC were transfected with 400 pmol siCtrl or siSlp2 for 48 h, and again with the same amount of the respective siRNA. 48 h after the second transfection, the anti-VWF antibody capture assay was performed as described in “Materials and methods” section, and levels of externalized and cell surface-localized VWF that captured anti-VWF antibodies present in the medium were quantified via flow cytometry. *N* = 8 experiments, significance tested with paired Student’s *t* test (**p ≤ 0.01). **B** Quantification of collagen-binding efficiency of secreted VWF after Slp2-a or Rab27a depletion, respectively. HUVEC were treated with the respective siRNAs as mentioned above for the VWF-ELISA and additionally analyzed by a type III collagen-ELISA. Collagen-binding capability (Act) was normalized to the total VWF released (Ag) in the respective conditions. Knockdown of Rab27a was used as a positive control. *n* = 6 experiments, significance tested with paired Student’s *t* test (**p ≤ 0.01). **C** HUVEC were transfected with equimolar amounts of GFP, Slp2a-GFP, or Slp2a-SHDonly-GFP Plasmids. 24 h Post-transfection, cells were stimulated with histamine or left untreated and the anti-VWF antibody capture assay was performed as described in “Materials and methods” section, and levels of externalized and cell surface-localized VWF that captured anti-VWF antibodies present in the medium were quantified via flow cytometry. *N* = 5 experiments, significance tested with ANOVA and Dunnett’s post hoc test. (ns = not significant, **p ≤ 0.01)

Francis et al. could show recently that Slp2-a is an upstream regulator of WPB exocytosis in the course of angiogenic lumen formation and that Slp2-a knockdown reduces stimulus-evoked VWF secretion [33]. Extending these findings, our analysis of VWF secretion after Slp2-a depletion (Fig. S3) revealed a reduction of both, the histamine-triggered release of VWF to the cell culture medium and the amount of exocytosed VWF appearing on the cell surface, which was detected using an anti-VWF antibody capture assay [11, 41] (Fig. 5A). Interestingly, though being statistically significant this reduction was rather modest as also observed before by analyzing the amount of intracellular VWF after stimulation of Slp2-a depleted cells [33]. Several other Rab27a effectors are known to be recruited to WPB and to regulate WPB exocytosis, in particular MyRIP and Slp4-a [10, 17]. Therefore, we also analyzed whether their WPB association is affected by Slp2-a knockdown which could result in indirect effects on WPB exocytosis. However, the localization of MyRIP and Slp4-a to WPB is not affected in a significant manner in Slp2-a depleted cells (Fig. S4). As the above secretion assays do not differentiate between low- and high-molecular-weight VWF multimers, we also performed type III collagen-binding assays which preferentially detect high-molecular-weight multimers among the VWF released into the cell culture supernatant [42–44]. Figure 5B shows that the type III collagen-binding capability of VWF secreted following histamine stimulation was significantly reduced in Slp2-a knockdown conditions as compared to control siRNA-treated cells. A similar reduction in VWF-collagen binding was observed upon Rab27a knockdown, which had been shown before to result in the release of less multimeric VWF [17]. This suggests that Slp2-a promotes exocytosis of fully matured WPB that contain highly multimeric VWF and are characterized by the rod-like morphology.

We next attempted to interfere with Slp2-a function by overexpressing the SH domain as this construct is recruited to WPB (Fig. 2) and likely acts in a competitive, dominant-negative manner. Figure 5C shows that expression of Slp2a-SHDonly-GFP leads to a modest but significant reduction in the histamine-evoked release of VWF, the extent of this reduction being similar to that observed upon Slp2-a knockdown. In contrast, overexpression of full-length Slp2a-GFP has no significant effect.

Together these data suggest that by tethering mature WPB via their tips to the PM, Slp2-a likely affects only this WPB population, and thus, Slp2-a depletion or SHD overexpression causes an only a modest effect on the total evoked VWF release. Interestingly, in gastric-surface mucous cells, Slp2-a has also been linked to the PM targeting of mucus-containing secretory granules but appears to be largely dispensable for evoked mucus secretion [35].

**Fig. 6:**
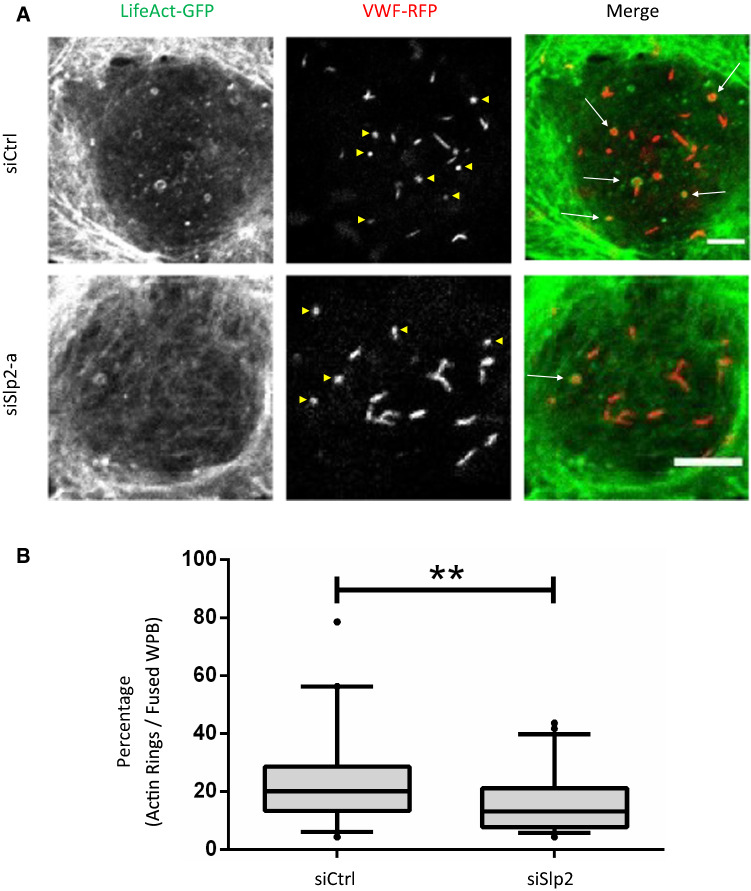
Loss of Slp2-a reduces the appearance of actin rings at fusing WPB. **A** HUVEC were transfected with 400 pmol of siSlp2 or siCtrl for 48 h, and transfected again with the same amounts of the respective siRNAs, LifeAct-GFP and VWF-RFP. 24 h after the second transfection, cells were subjected to time-lapse confocal microscopy and stimulated with 500 µM histamine during acquisition. One cell was imaged for approximately 5 min. Example image shows stills of one plane of the respective cell after stimulation with histamine. Actin rings (green; some highlighted by arrows) can be seen as circles around VWF fusion spots (red). Scale bar = 5 µm. Yellow arrowheads indicate fusion spots of WPB **B** Quantification of the percentage of actin rings per fused WPB. WPB (VWF-RFP) were counted before stimulation and at the last time-point of image acquisition (after histamine stimulation) to obtain the number of fused WPB. Actin rings around VWF fusion spots were also counted and are expressed as a percentage relative to the total number of fused WPB. Boxplot with 5–95% confidence interval, with black dots as outliers. *n* ≥ 39 cells of 5 different experiments, significance tested with unpaired Mann–Whitney test. (**p ≤ 0.01). The percentage of visible actin rings at WPB fusion sites (appr. 20%) is in line with previous experiments carried out under these histamine conditions [29]. Very transient or weak actin rings are probably not detected

A potential role of Slp2-a in linking the tips of WPB to the PM should reflect itself in the alteration of structures that depend on such fusion topology. Rings or coats of polymerized actin have been shown to form at the base of WPB following fusion with the PM and these actin rings in conjunction with myosin motor activity have been reported to support the expulsion of highly multimeric VWF from fused WPB by driving a movement of the fused granule toward the PM [31]. Such activity would benefit from a tip-end fusion topology, and therefore, we analyzed the formation of actin rings in live HUVEC by employing Lifeact-GFP as marker. Depletion of Slp2-a significantly reduced the number of actin rings at WPB–PM fusion sites supporting the view that Slp2-a fosters a tip-end fusion topology as prerequisite for the assembly of actin rings at the distal end of fused WPB, which in turn support the expulsion of highly multimeric VWF (Fig. 6).

### PI(4,5)P_2_-rich PM domains likely function as Slp2-a interaction sites

Slp2-a is known to interact with the negatively charged lipids PI(4,5)P_2_ and PS [34]. Interestingly, PI(4,5)P_2_ has recently been shown to accumulate at WPB–PM fusion sites and specific depletion of PI(4,5)P_2_ in the plasma membrane resulted in compromised secretagogue-stimulated WPB exocytosis and VWF secretion [27]. In contrast, PS shows no specific accumulation at WPB–PM fusion sites but appears to be slightly enriched in the membrane of resting WPB with this enrichment being lost upon histamine stimulation [27]. Because of these dynamic changes in the distribution of the Slp2-a binding lipids PI(4,5)P_2_ and PS in the course of secretagogue-evoked WPB exocytosis, we next investigated the influence of Slp2-a lipid binding on its tip localization in a more specific manner exploiting mutations in the C2A domain of Slp2-a that interfere with lipid binding [34]. Slp2a-KQ-C2A, in which 5 lysines within the C2A domain that are essential for binding of negatively charged lipids are mutated to glutamines, completely lacks binding to either PI(4,5)P_2_ or PS. In Slp2a-DN-C2A, in which the aspartates critical for Ca^2+^ binding are mutated to asparagines, the binding of PI(4,5)P_2_ is unchanged, while the binding of PS is no longer dependent on the Ca^2+^ concentration [34].

**Fig. 7:**
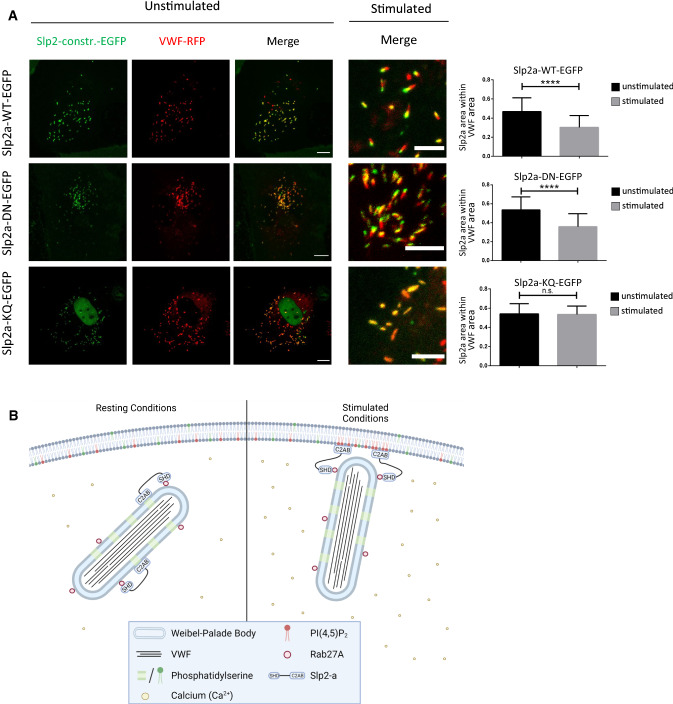
Point mutations that change the lipid-binding profile of the C2AB domain interfere with Slp2-a tip localization. **A** Left: HUVEC transfected with the indicated Slp2-a constructs and VWF-RFP were subjected to time-lapse confocal microscopy and stimulated with 500 µM histamine during acquisition (see also Videos 6 and 7). Stills of the stimulated conditions represent time-point after a few WPB had undergone fusion. Scale bar: 10 µm for unstimulated overviews, 5 µm for stimulated close-ups. Right: Tip localization was quantified as described in “Materials and methods” section from *n* ≥ 12 cells; error bars = SD. Significance was tested with paired Student’s *t* test (****p ≤ 0.0001). **B** Model depicting the possible function of Slp2-a in WPB exocytosis. Rab27a recruits Slp2-a to WPB via its SHD. In resting conditions, the C2AB domain of Slp2-a binds to phosphatidylserine (PS) on the membrane of WPB. PS binding by Slp2-a is efficiently inhibited by Ca^2+^ concentrations within the physiological range for exocytosis events, while PI(4,5)P_2_ binding is largely unaffected by Ca^2+^. After histamine stimulation and the resulting rise in intracellular Ca^2+^, the PS binding thus is weakened, which might allow WPB-resident Slp2-a to bind PM-resident PI(4,5)P_2_ via the now available C2 domain. As a consequence, Slp2-a would be enriched at that part of the WPB which is closest to the PM and permits interactions with PM PI(4,5)P_2_. Additional PI(4,5)P_2_ might be laterally recruited toward the future site of exocytosis, and the interaction between organelle and plasma membrane would be strengthened. By switching from PS to PI(4,5)P_2_-binding in a Ca^2+^-dependent manner, Slp2-a would thus ensure sufficient proximity between WPB tips and the PM to favor subsequent fusion in this topology. Figure created with BioRender.com

We expressed the respective DN and KQ mutants of Slp2-a as GFP fusions in HUVEC and analyzed their dynamic distribution by live-cell confocal microscopy. As seen for Slp2a-wt-GFP, both mutants localized to VWF-RFP positive WPB in resting HUVEC with cells expressing Slp2a-DN-GFP often displaying a clustering of WPB in the cell periphery (Fig. 7). Histamine treatment of HUVEC expressing the DN and KQ mutants triggered abundant WPB–PM fusions, suggesting that neither mutant exhibited a strong dominant-negative phenotype on evoked WPB exocytosis at the expression level achieved here. We did not attempt to systematically quantify WPB–PM fusions within our confocal dataset to avoid ambiguity due to out-of-focus events. Moreover, the tight clustering of WPB in Slp2a-DN-GFP overexpressing cells precluded the identification of single fusion events in many cases. However, we were able to study tip localization—or the lack thereof—of the Slp2-a mutants, precisely because WPB fusions still took place. Compared to Slp2a-wt-GFP, the propensity for tip localization in Slp2a-DN-GFP seems normal (Fig. 7, Video 6). In stark contrast, we observed no evidence of tip localization of Slp2a-KQ-GFP after histamine stimulation (Fig. 7, Video 7). Together, these data indicate that tip localization of Slp2-a requires lipid binding via the C2A domain of Slp2-a, most likely involving an interaction of Slp2-a with PM PI(4,5)P_2_ that is enriched at WPB–PM fusion sites.

To further substantiate a potential role of PM PI(4,5)P_2_ in mediating Slp2-a tip localization, we analyzed the Slp2-a behavior in cells depleted of phospatidyl 4-phopshate 5-kinase γ (PI4P5Kγ), the major enzyme generating PI(4,5)P_2_ in HUVEC. We had shown before that this depletion and a reduction in PM PI(4,5)P_2_ interfere with histamine-evoked WPB exocytosis [27]. Interestingly, PI4P5Kγ knockdown also results in a significant reduction in the extent of Slp2-a tip localization after histamine stimulation (Fig. S5). This supports the hypothesis that PM PI(4,5)P_2_ participates in promoting Slp2-a tip localization prior to WPB–PM fusion.

## Discussion

In this work, we investigated the possibility that an elongated, rod-shaped organelle, here the WPB of endothelial cells, can initiate fusion with the PM preferentially at one of its tips. We present evidence that the frequency of tip fusions outweighs that of strictly lateral fusions, at least for histamine-stimulated conditions. In search for factors potentially mediating this tip-end (or polar) fusion, we identify Slp2-a as a WPB-associated protein that becomes enriched at the organelle tip which will undergo fusion. Slp2-a, the depletion of which decreases the extent of histamine-induced, Ca^2+^-dependent secretion of highly multimeric VWF, is a PI(4,5)P_2_ and PS-binding protein. Its phospholipid-binding sites are located in the Ca^2+^ regulated C2A domain and we show that this domain, and in particular its PI(4,5)P_2_/PS-binding lysine residues, is required for Slp2-a tip localization following histamine stimulation. As PI(4,5)P_2_ but not PS accumulates at WPB–PM fusion sites and its depletion interferes with Ca^2+^-regulated WPB exocytosis [27], we interpret these data to indicate that Slp2-a is instrumental in establishing contact between PM PI(4,5)P_2_ and the tips of fusing WPB. Thus, Slp2-a is required for efficient tip-end fusion and beneficial for the release of highly multimeric VWF, but not for WPB–PM fusion per se explaining the relatively mild inhibitory effect of Slp2-a knockdown on total VWF secretion.

Why do WPB preferentially initiate fusion at one of their tips? On one hand, high membrane curvature is beneficial for efficient fusion [45]. The nearly hemispherical tips of WPB represent the most highly curved membrane regions of the organelle, and a local accumulation of Slp2-a on the pre-fusion WPB tip would serve to further functionalize this membrane region. On the other hand and apart from biophysical considerations, tip fusion conceivably has advantages for cargo release. VWF expulsion from WPB is regulated by the de-acidification of the organelle lumen [46], and VWF tubules are “spring-loaded” for cargo release upon de-acidification of the organelle lumen following WPB–PM fusion [47]. The resulting “unspooling” of VWF tubules that lead to VWF string formation in the blood vessel is more naturally accomplished along the length of VWF tubules present in a tip-fused configuration. An alternative or additional mechanism for VWF release provided by a contractile actin ring around WPB expelling VWF by actomyosin-driven forces is also reconciled more easily with tip fusions rather than lateral cargo release as the actin ring has been shown to form at the base of a (tip) fused WPB and appears to push the fused elongated granule toward the plasma membrane [31]. In line with these considerations and the requirement of Slp2-a for tip-end fusion, Slp2-a knockdown reduces the number of actin rings at WPB–PM fusion sites and the amount of highly multimeric VWF released following histamine stimulation. Apart from supporting efficient release of large multimeric VWF, tip fusion would also benefit kiss-and-run and cavicapture type fusion events of the large asymmetric WPB. Furthermore, it also is possible that tip fusion regulates the release of (unknown) factor(s) that are concentrated at one end of mature WPB.

How is the Slp2-a accumulation at the WPB tip which will undergo fusion achieved? While Slp2-a recruitment to WPB depends on Rab27a, stimulation-dependent tip localization is only undergone by Slp2-a itself, and not by Rab27a. We think that it is likely that Ca^2+^-dependent changes in Slp2-a/lipid interactions at the PM and also the organelle membrane are the driving factor in stimulus-induced Slp2-a tip localization. PS binding by Slp2-a is efficiently inhibited by Ca^2+^ concentrations within the physiological range for exocytosis events, while PI(4,5)P_2_ binding is largely unaffected by Ca^2+^ [34]. As PS is slightly enriched in the WPB membrane of resting but not histamine-stimulated HUVEC [27], we speculate that Slp2-a binds via its C2A domain to PS on the WPB membrane in resting HUVEC at low intracellular Ca^2+^ concentrations. After histamine stimulation, this PS binding would be weakened, which might allow the C2A domain of WPB-localized Slp2-a to bind PM-resident PI(4,5)P_2_ instead (Fig. 7B). As a consequence, Slp2-a would be enriched at that part of the WPB which allows for interactions with PM PI(4,5)P_2_. Additional PI(4,5)P_2_ and/or phosphatidic acid (PA), another anionic phospholipid known to accumulate at WPB–PM fusion sites [27] might be laterally recruited toward the future site of exocytosis, and the interaction between organelle and plasma membrane would be strengthened. By switching from PS to PI(4,5)P_2_-binding in a Ca^2+^-dependent manner, Slp2-a would thus ensure sufficient proximity between WPB tips and the PM to favor subsequent fusion in this topology. In this model, the WPB tip that develops the Slp2-a accumulation would simply be the one in closest proximity to the plasma membrane, and in consequence, closest to a source of PI(4,5)P_2_. We have currently no indication that the two tips of any single WPB are inherently different from each other before Slp2-a accumulation, apart from their relative positions within the cell. This model, however, does not fully explain the behavior of the Slp2a-DN mutant which accumulates at WPB tips after histamine stimulation albeit apparently not being able to respond to rising Ca^2+^ concentration by a weakening of the PS binding. We speculate that the PI(4,5)P_2_ binding of this mutant is so dominant that it can still promote tip localization despite an unaltered PS interaction. As an alternative to PM lipids (PI(4,5)P_2_, PA) promoting tip localization of Slp2-a, the higher curvature at WPB tips or a yet to be identified factor recruited specifically to WPB tips could participate in mediating Slp2-a enrichment. However, recruitment of such factor would have to be stimulus-dependent as Slp2-a localizes over the entire WPB in resting, unstimulated HUVEC.

With MyRIP, Slp4-a, Munc13-4, and Slp2-a all affecting VWF secretion from endothelial cells, the involvement of Rab27a effectors in WPB exocytosis has now reached a certain level of complexity. However, not all of these act as Rab27a effectors exclusively: Slp4-a is initially recruited to WPB by Rab3 [10], and Rab27a is dispensable for Munc13-4 recruitment to WPB [14, 39]. Nevertheless, the question arises why so many Rab27a effectors are involved in this process. A potential explanation may lie in the fact that while endothelial cells are apico-basally polarized in vivo, most experiments addressing the molecular mechanisms of WPB exocytosis so far have been performed in non-polarized cell culture systems. One exception is a study that revisited the long-standing issue of how strongly VWF secretion is polarized, and found the regulated release of VWF to occur predominantly apically, i.e., toward the blood vessel lumen [48]. Intriguingly, Slp2-a was described earlier to not only act in establishing apico-basal polarity within renal epithelial cells [38, 49], but also to be involved in targeting fusiform vesicles, another class of large, curved secretory vesicles, toward the apical domain of urothelial umbrella cells [50]. Importantly, in endothelial cells, a recent study could show that Slp2-a supports the WPB-mediated apical secretion of angiopoietin-2 [33]. In MDCK II cells, Slp2-a expression is strongly upregulated in 3D differentiated cells compared to non-polarized growth conditions. In this respect, it is interesting to note that we also observed an upregulation of Slp2-a mRNA expression when HUVEC reach confluency in culture, i.e., when a first apico-basal polarity is established (Fig. S6). A specific role of Slp2-a in apical VWF secretion is also supported by the involvement of PI(4,5)P_2_, which is enriched in the apical PM of epithelial cells [51]. Moreover, in Jurkat cells, an interdependence between Rab27a/Slp2-a protein abundance and PI(4,5)P_2_ content of the plasma membrane has been found in the context of secretion of HIV particles [52]. Thus, Slp2-a likely functions in conjunction with PI(4,5)P_2_-rich PM domains in supporting a unique tip-end tethering and eventually fusion of WPB, thereby permitting a regulation of selective WPB cargo release.

## Materials and methods

### Cell culture and transfection

HUVEC were obtained from Promocell and cultured in a mixed endothelial growth medium, a 1:1 mixture of ECGM-2 (C-22111, Promocell) and M199 (F0615, Biochrom) + 10% fetal calf serum (Sigma) + 100 i.u. heparin (Ratiopharm), supplemented with 0.015 µg/ml amphotericin B (Biochrom) and 30 µg/ml gentamycin (CytoGen), at 37 °C and 5% CO_2_ for up to five passages. HUVEC were transfected with plasmids or siRNAs using the Amaxa Nucleofection Technology (HUVEC Nucleofector Kit-OLD, Lonza) as described previously [13]. Transfections with plasmid DNA used 1–10 µg DNA per 20 cm^2^ of nearly confluent HUVEC. VWF-EGFP [53], VWF-RFP [54], and Lifeact-GFP [55] were described before and kindly provided by Tom Carter (St. George University, London) and Roland Wedlich-Söldner (University of Münster), respectively. Rab27a-EGFP was generated by amplification of the Rab27a cDNA from a HUVEC cDNA library using specific primers that included EcoRI and BamHI restriction sites at the 5′ and 3′ end, respectively, and cloning of the PCR product into the pEGFP-C1 vector (Clontech).

- Rab27F1: CCGGAATTCAATCTCTGATGGAGATTATG
- Rab27R: CGCGGATCCTCAACAGCCACATGCCCCTT

Rab27a-strby was then generated by exchanging the fluorophore of Rab27a-EGFP, using pmStrawberry (Clontech) as the fluorophore source and specific primers with the restriction sites AgeI and BamHI at the 5′ and 3′ end, respectively. Slp2a-wt-GFP, Slp2a-ΔC2AB-GFP, Slp2a-ΔSHD-GFP, Slp2a-SHDonly-GFP, Slp2a-E11A/R32A-GFP, and Slp2a-V18A-GFP have been described [38]. Slp2a-DN-GFP and Slp2a-KQ-GFP were generated by site-directed mutagenesis to match the single amino acid changes described in [34] using the Q5 site-directed mutagenesis kit (New England Biolabs) according to the manufacturer’s instructions in two rounds of mutagenesis each using the following primers:

- KQ1: CAAAGGCAAAATGGGCCAGCAGCAAACACTCGTAGTGAAGAAAAC
- KQ2: GCAGCAAACACTCGTAGTGCAGCAAACCTTGAATCCTGTG
- DN1: CTTAGCAGCAGCGAATGTAAAAAAACAGCGTTCAAACCCATATGTAAAGG
- DN2: GAACCTGTCCATTTGGCATCGGAATACATTTAAGCG.

Transfections with siRNAs used 400 pmol targeting or control siRNA per 20 cm^2^ dish of nearly confluent HUVEC. Rab27a was depleted using the siRNA 5′GGAGAGGUUUCGUAGCUUA-dTdT (Sigma), and Slp2 with siSlp2 5′CGUUCAGACCCAUAUGUAA-dTdT (Microsynth). For control conditions, AllStars Neg. Control siRNA (siCtrl) (102,781, Qiagen) was used. After incubation for 48 h, siRNA transfection was repeated with or without any additionally required plasmid DNA. Cells were used for experiments 48 h after the second round of transfection.

Knockdown of PI4P5Kγ was performed as described by Nguyen et al. [27]. Inhibition of Exo70 with Endosidin2 was performed by treating HUVEC for 2 h at 37 °C and a final concentration of 10 µM [40].

### Microscopy

HUVEC transfected with the appropriate constructs were seeded in 8-well glass bottom µ-slides (ibidi) pretreated with collagen type I from rat tail (Millipore). During acquisition, cells were maintained in a mixed endothelial growth medium containing 20 mM HEPES at 37 °C in the incubation chamber of the respective microscope. Histamine stimulation during acquisition was performed manually by adding 100 × concentrated histamine stock solution to final concentrations ranging from 20 to 500 µM as indicated in the respective figure legends.

Confocal microscopy was performed on an LSM780 confocal system (Zeiss) using a 63 × , NA 1.4 oil immersion objective (Plan-Apochromat, Zeiss) or on an LSM800 confocal system (Zeiss). TIRF microscopy with simultaneous epifluorescence illumination was performed on an Olympus IX71 TIRF Microscope customized to include a heated incubation chamber, an objective-type TIRFM setup from TILL Photonics, a monochromator for epifluorescence excitation, and a controller allowing hardware-controlled fast switching between total internal reflection fluorescence and epifluorescence (TILL Photonics). Images were acquired using a TILL Image QE charge-coupled device camera (TILL Photonics) and MetaMorph Software (Molecular Devices). The total internal reflection angle was manually adjusted for every experiment. TIRF movies were recorded with 2–5 frames per second. Image analysis for fusion mode (tip vs. lateral fusion) was done in Fiji [56, 57].

Preparation of microscopic images for figures was performed in Fiji using linear brightness/contrast adjustments only. For assessment of Slp2a-GFP tip localization on WPB, two time-points were chosen in the recording of each histamine stimulation experiment analyzed: (1) the first, pre-stimulation image, and (2) a time-point post-stimulation at which some, but not all WPB fusions had occurred. To compare the occupancy of Slp2a-GFP on VWF-RFP-positive objects before and after stimulation, each image was processed as follows: First, each bicolor image was split into its constituent channels; then, object segmentation was performed on the VWF-RFP channel by manually adjusting and applying a threshold, creating a binary mask (Image—> adjust—> threshold—> manually select—> apply; edit—> selection—> create mask). The mask was then loaded into the ROI manager (edit—> selection—> create selection; analyze—> tools—> ROI manager—> add). To segment the WPB in the Slp2a-GFP channel, the VWF-RFP mask was then applied to the Slp2a-GFP channel (select green channel image—> select VWF-RFP ROI in ROI manager- > edit—> clear outside; image—> adjust threshold—> apply; edit—> selection—> create mask), and subsequently loaded to the ROI manager as well. With these tools and the command “measure” in the ROI manager, the area occupied by the VWF signal in the VWF-RFP channel, and the area covered by the Slp2a-GFP signal within the VWF-RFP mask were measured each. The area representing Slp2a-GFP occupancy on WPB was then expressed as a percentage of the VWF-RFP mask representing the entire WPB to compare pre-stimulation and post-stimulation time-points. The statistical significance of results was assessed using the unpaired *t* test in GraphPad Prism.

To microscopically analyze the formation of actin rings at WPB–PM fusion sites, one cell per well was recorded. The cell was divided into 8 z-stacks that were recorded one after another. After completion of the first complete z-stack, histamine was added to a final concentration of 500 µM and microscopy was performed for approximately 5 min per cell. Statistical significance of results was assessed using the unpaired Mann–Whitney test in GraphPad Prism.

### Immunofluorescence staining

HUVEC were seeded on collagen-coated 12 mm glass coverslips and cultivated at 37 °C, 5% CO_2_. Cells were fixed in 4% PFA in 1 × PBS for 10 min at RT and subsequently permeabilized using 0.1% Triton X-100 for 2 min. After blocking (3% BSA in 1 × PBS) for 30 min, primary antibodies were applied at the dilutions indicated. Cells were washed and treated with secondary antibodies and DAPI, if applicable. After the final washing, HUVEC were mounted on glass slides using Mowiol. Samples were analyzed using a confocal laser scanning microscope LSM800 (Zeiss). Primary antibodies used: rabbit-anti-Slp4-a, Atlas Antibodies; ms-a-VWF, DAKO; goat-anti-MyRIP, Abcam.

### Western blotting and antibodies

HUVEC lysate was prepared by harvesting cells from a 20 cm^2^ dish with trypsin/EDTA, washing the cell pellet in PBS once, and lysing the cells by sonication for 1 min in 40 μl lysis buffer (20 mM HEPES, 150 mM NaCl, 0.5% Triton X-100, 1.5 mM PMSF and complete EDTA-free protease inhibitor cocktail (11,873,580,001, Roche) added according to the manufacturer’s instructions). After incubation on ice for 15 min, cellular debris was pelleted (10 min, 1250 × g, 4 °C) and the cleared lysate was subjected to SDS-PAGE and Western blotting according to the standard protocols. For characterization of Rab27 effectors expressed in HUVEC, 40 µg of the lysate was loaded per lane and compared to T7-tagged proteins expressed in COS7 cells. SDS-PAGE was performed in 7.5% acrylamide gels, and Western blotting was carried out as described with primary antibodies generated previously [58, 59]. For characterization of Slp2a-GFP knockdown efficiency, the primary antibodies used were mouse anti-β actin mAB, clone AC-15 (Sigma-Aldrich) (loading control), and rabbit anti-GFP pAB (Invitrogen). The secondary antibodies used were goat anti-rabbit IRDye 680 (LICOR) and goat anti-mouse IRDye 800 (LICOR), imaged with an Odyssey Infrared Imaging System (Application Software version 3.0, LICOR).

### Secretion assay

HUVEC grown to full confluency were used for all secretion assays. Stimulation was performed with 100 µM histamine (Sigma-Aldrich). SiRNA-treated HUVEC were used approx. 48 h after the second round of transfection. The amount of secreted VWF was quantified by enzyme-linked immunosorbent assay (ELISA) as previously described [18], analyzing three triplicate samples per condition (supernatant of unstimulated cells, of stimulated cells, and total lysates). The amount of VWF secreted into the cell culture supernatant was then expressed as percentage of total VWF content (amount of VWF of all three samples combined, that is, supernatant of nonstimulated plus supernatant of stimulated cells plus remaining total lysate). This analysis allowed for controlling for differences in total VWF content between each replicate and each condition. The collagen-binding capability of secreted VWF was determined using a well-established collagen-ELISA, in addition to the described VWF-ELISA [42, 43]. In brief, ELISA plates were coated with human type III collagen (Southern Biotech) and then processed similar to the VWF-antigen-ELISA. Collagen-binding ratio was calculated by evaluating the ratio between collagen-binding and VWF antigen levels. Secretion assays in which control stimulation reached < 18% were excluded from the analysis. Statistical significance was assessed with paired *t* tests in SPSS or in GraphPad Prism.

### Flow cytometry-based capture assay

HUVEC grown to full confluency were used for all capture assays. Stimulation was performed with 100 µM histamine (Sigma-Aldrich). SiRNA-treated HUVEC were used approx. 48 h after the second round of transfection. GFP transfected HUVEC were used approx. 24 h after transfection. HUVEC were treated with rabbit-anti-VWF antibodies (DAKO) for 20 min in basal medium (2% (w/v) BSA in M199) to saturate non-specific binding sites. Cells were then washed and treated with rabbit-anti-VWF DyLight™ 650 antibodies with or without stimulant (histamine), for 20 min in a basal medium. Subsequently, HUVEC were washed and deattached from the dish using Accutase (PAA). After centrifugation (4 min, 4 °C, 200*g), cells were washed and then fixed and permeabilized by applying Fixation/Permeabilization solution (BD Cytofix/Cytoperm™) for 20 min. Cells were then blocked (BD Perm/Wash™ Buffer) for 15 min. For staining of total VWF, HUVEC were treated with rabbit-anti-VWF DyLight™ 405 antibodies for 1 h, washed three times with 5% BSA (w/v) in 1xPBS, and subsequently analyzed. Per condition 10.000 cells (in case of GFP constructs 10.000 GFP expressing cells) were counted and analyzed for their surface VWF (DyLight^™^ 650) and total VWF (DyLight^™^ 405) signal intensities on a Guava^®^ easyCyte^™^ Flow Cytometer. The statistical significance of results was assessed using the *t* test or ANOVA with Dunnett's post hoc test in GraphPad Prism.

### Real-time PCR

RNA from HUVEC was isolated using RNEasy Mini Kit (Qiagen). For cDNA Synthesis 1 µg of RNA was transcribed using High-Capacity cDNA Reverse-Transcription Kit (Thermo Fisher Scientific). Real-time PCR was performed with Brilliant III Ultra-Fast SYBR^®^ Green qPCR Mastermix (Agilent) on a Roche LightCycler 480 according to the manufacturer’s instructions. Analysis was performed using the ${2}^{-\Delta \Delta {\mathrm{C}}_{\mathrm{T}}}$-Method [60]. The following primers were used:

- VWF-fw: CCT TGA ATC CCA GTG ACC CTG A
- VWF-rev: GGT TCC GAG ATG TCC ACA T
- Slp2: Hs_SYTL2_1_SG QuantiTect Primer Assay QIAGEN (Cat. No. QT01021629)
- ACTB: Hs_ACTB_1_SG QuantiTect Primer Assay QIAGEN (Cat. No. QT00095431)
- B2M: Hs_B2M_1_SG QuantiTect Primer Assay QIAGEN (Cat. No. QT00088935)

Housekeeping genes: β-Actin (ACTB) and β2-Mikroglobulin (B2M). Statistical significance of results was assessed using the unpaired *t* test or Mann–Whitney test in GraphPad Prism.

### Statistics

All statistical analyses were performed using GraphPad Prism or IBM SPSS Statistics. Asterisks mark statistically significant results: ****p ≤ 0.0001, ***p ≤ 0.001, **p ≤ 0.01, *p ≤ 0.05. Normal distribution was assessed by the Shapiro–Wilk test, *p* < 0.05. Normally distributed data were analyzed employing Student’s *t* test or one-way ANOVA with Dunnett’s post hoc test (> 2 conditions). Non-parametric data were analyzed using Mann–Whitney test.

## Supplementary Information

Below is the link to the electronic supplementary material.

Supplementary file1 (PDF 2882 KB)

Supplementary file2 (MP4 1303 KB)

Supplementary file3 (MP4 29 KB)

Supplementary file4 (MP4 2173 KB)

Supplementary file5 (MP4 6720 KB)

Supplementary file6 (MP4 229 KB)

Supplementary file7 (MP4 252 KB)

Supplementary file8 (MP4 770 KB)

## Data Availability

The datasets generated during and/or analyzed during the current study are available from the corresponding author (gerke@uni-muenster.de) on reasonable request.
