# Supplementary material for: Tip-end fusion of a rod-shaped secretory organelle
Source: Cell Mol Life Sci. 2022 Jun 4;79(6):344. doi: 10.1007/s00018-022-04367-2 (PMC9167223; doi:10.1007/s00018-022-04367-2)
Supplement: Supplementary file 1 — Supplementary file1 (PDF 2882 KB) [file 18_2022_4367_MOESM1_ESM.pdf]

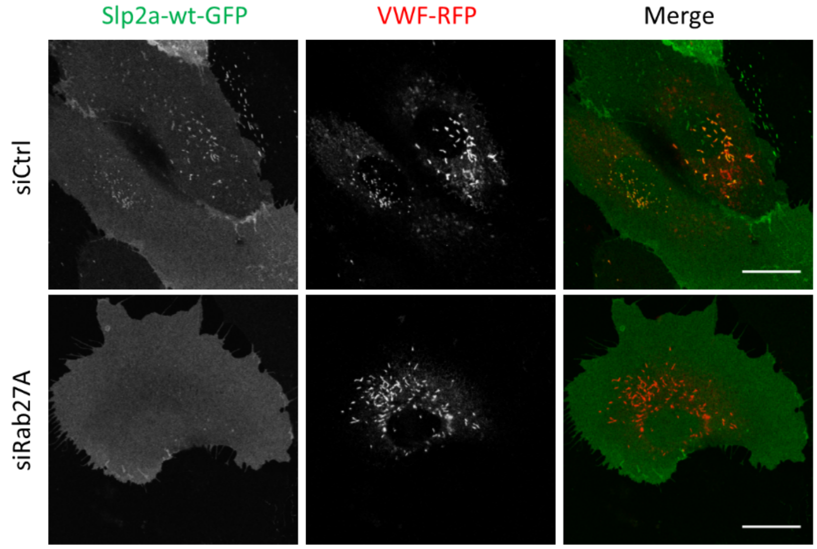

Figure S1. WPB association of Slp2-a requires Rab27a

HUVEC were treated with siCtrl or siRab27 and transfected with Slp2a-wt-GFP and VWF-RFP. Shown are stills of the respective live cell recordings. Scale bars = 20 µm

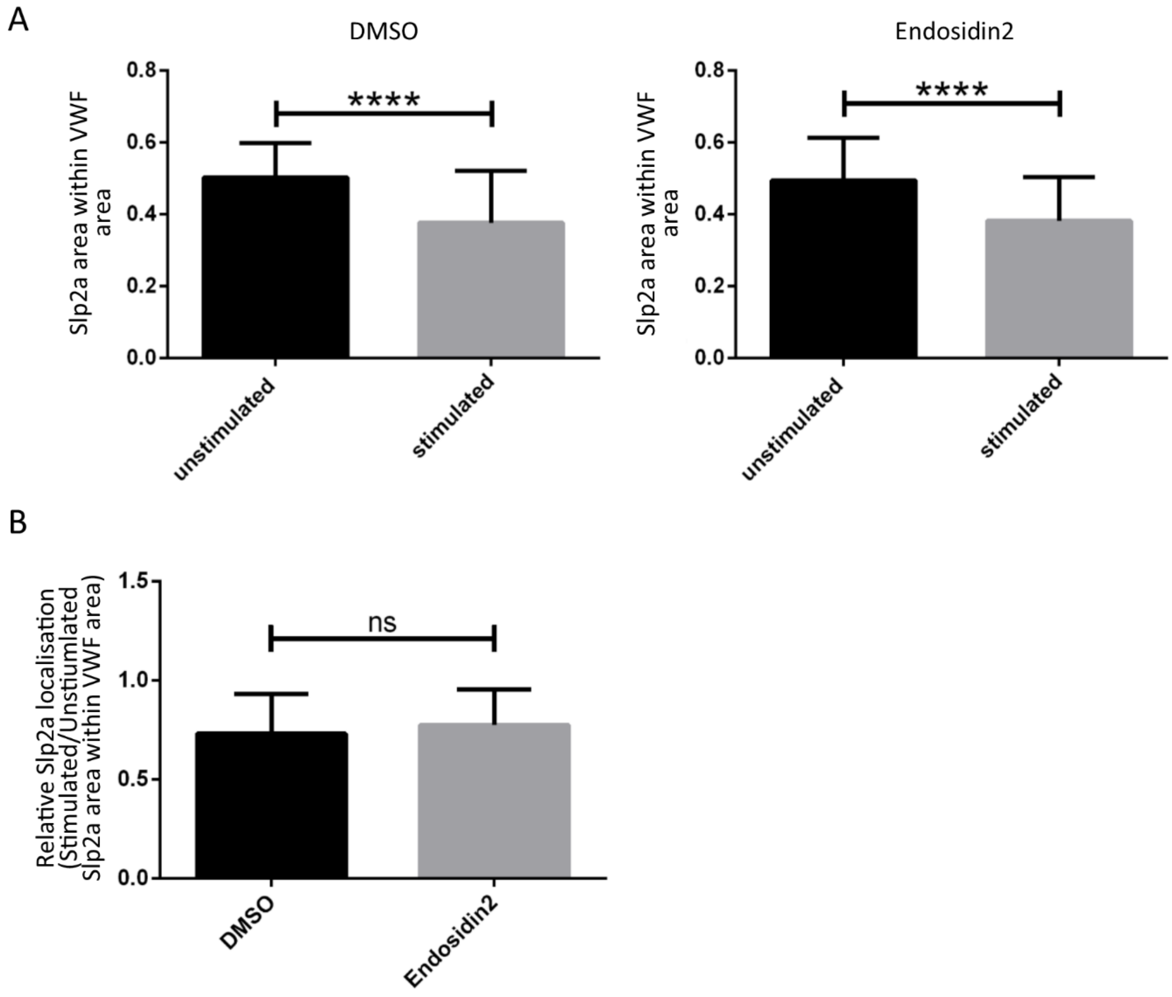

Figure S2. Exocyst inhibition by Endosidin2 has no effect on Slp2-a tip localization

HUVEC were transfected with Slp2a-GFP and VWF-RFP. 22h after transfection cells were either treated with DMSO or Endosidin2 (10µM) for 2h. HUVEC were then subjected to time-lapse confocal microscopy and stimulated with 500 µM histamine during acquisition. **A.** Tip localization was quantified as described in Materials and Methods from n ≥ 19 cells from 2 different experiments; error bars = SD. Significance was tested with paired student's t-test (**** p ≤ 0.0001). **B.** Relative Slp2-a localization on WPB was calculated by dividing the overlapping area of Slp2-a with VWF in the stimulated condition by the respective value of the overlapping area of Slp2-a with VWF in the unstimulated condition. A value < 1 indicates less overlap of Slp2-a with VWF after stimulation and thus reveals tip localization, a value = 1 indicates no difference of overlap of Slp2-a with VWF after stimulation and a value > 1 indicates more overlap of Slp2-a with VWF after stimulation. N ≥ 19 cells from 2 different experiments; error bars = SD. Significance was tested with unpaired student's t-test (ns = not significant).

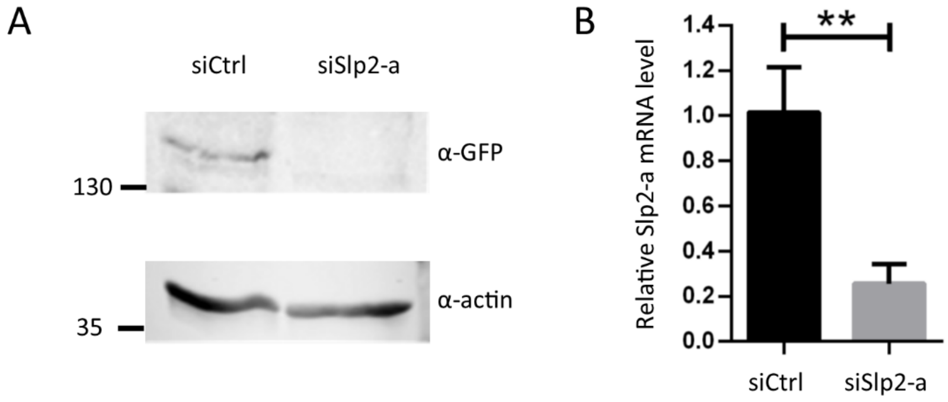

Figure S3. Validation of Slp2-a knockdown efficiency.

A. Left: Western blot analysis for assessment of Slp2-a knockdown efficiency. HUVEC were transfected with 400 pmol of siSlp2 or siCtrl, kept for 48 h, and then transfected again with the same amounts of the respective siRNAs and 3 µg of Slp2-a-GFP. Protein lysate was prepared 48 h after the second transfection, and the western blot membrane probed with anti-GFP and anti-actin antibodies as loading control. Left, molecular weight standards (kDa). **B.** Relative Slp2-a mRNA level in HUVEC. HUVEC were transfected with 400 pmol of siSlp2 and siCtrl for 48 h, and transfected again with the same amounts of the respective siRNAs. RNA was isolated 48h after the second transfection and analyzed via quantitative RT-PCR. n=4 experiments, significance tested with unpaired student's t-test (** $p \leq 0.01$).

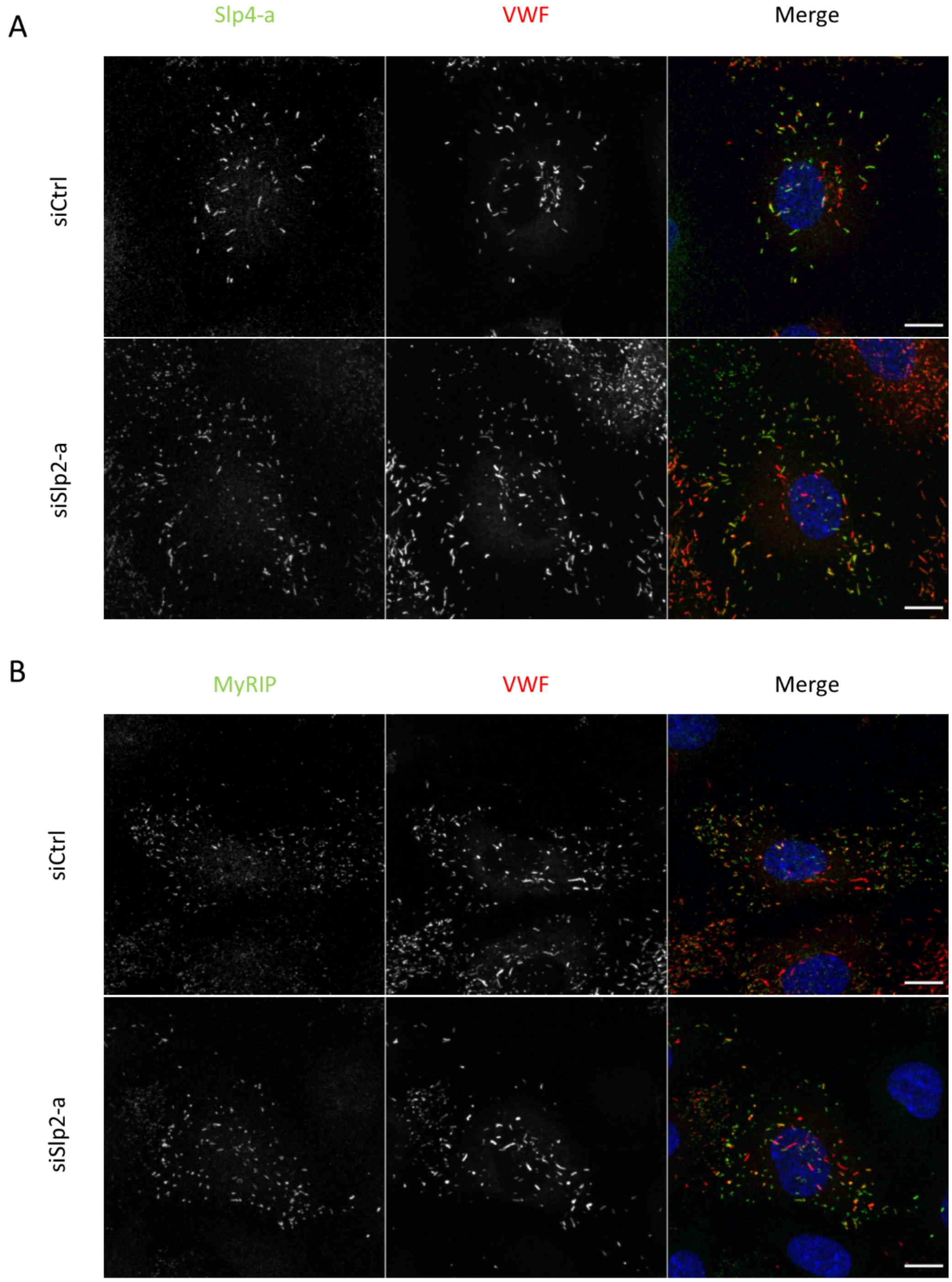

Figure S4. Slp2-a knockdown does not alter localization of other Rab27a effectors in HUVEC

HUVEC were transfected with 400 pmol siCtrl or siSlp2-a, kept for 48h, and were then again transfected with the same amount of the respective siRNA. Cells were fixed 48h after the second transfection and stained for VWF as WPB marker and **A.** Slp4-a or **B.** MyRIP using the respective antibodies. Samples were analyzed using a confocal laser scanning microscope.

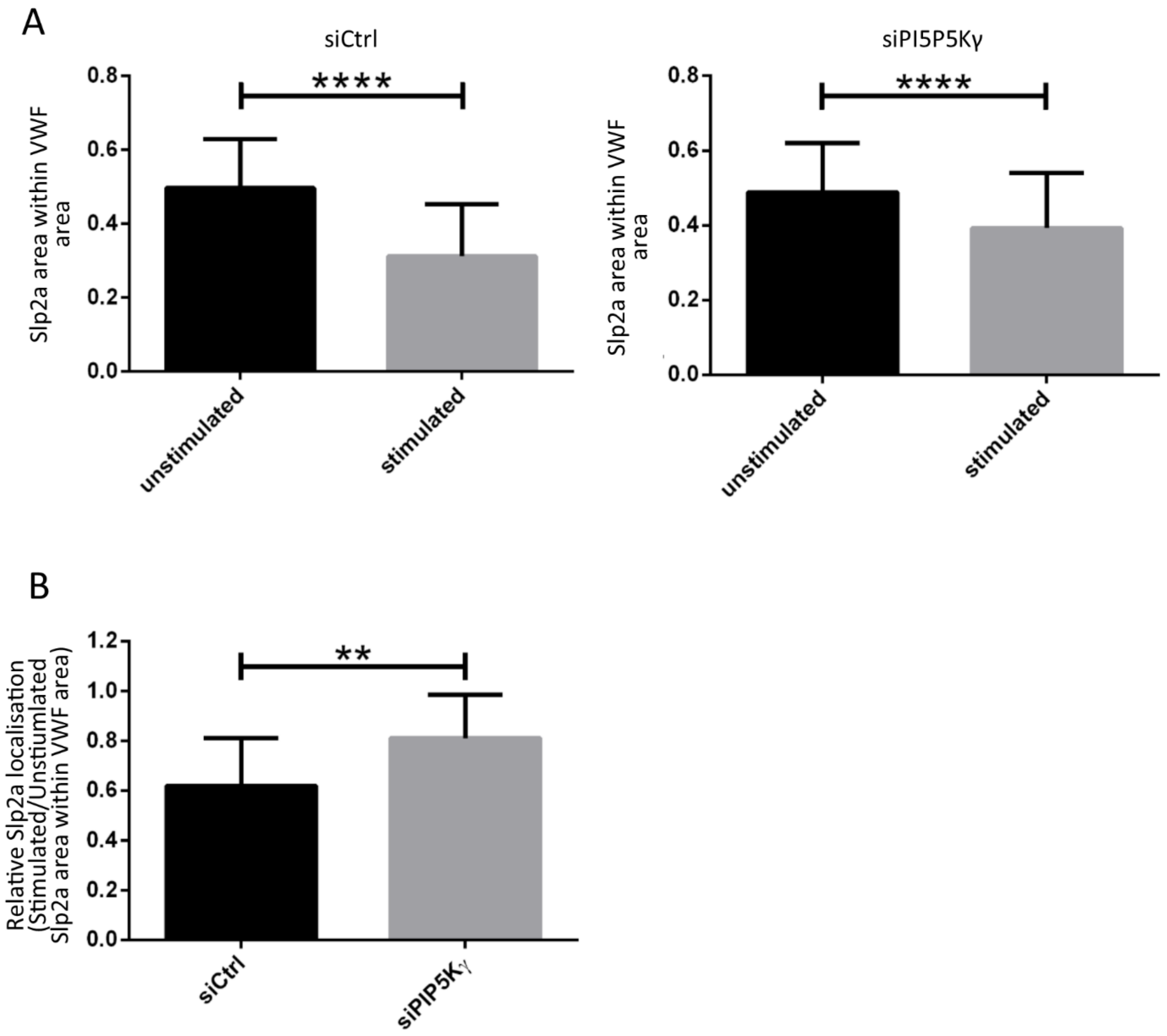

Figure S5. Knockdown of PI4P5Kγ reduces histamine induced Slp2-a tip localization

HUVEC were transfected with 100 pmol siCtrl or siPIP5Kγ, kept for 48 h, and then again transfected with the same amount of the respective siRNA, as well as Slp2a-GFP and VWF-RFP. Cells were subjected to time-lapse confocal microscopy 24h after the second transfection and stimulated with 500 µM histamine during acquisition. **A.** Tip localization was quantified as described in Materials and Methods from n = 21 cells of 3 different experiments; error bars = SD. Significance was tested with paired student's t-test (**** $p \leq 0.0001$). **B.** Relative Slp2-a localization on WPB was calculated by dividing the overlapping area of Slp2-a with VWF in the stimulated condition by the respective value of the overlapping area of Slp2-a with VWF in the unstimulated condition. A value < 1 indicates less overlap of Slp2-a with VWF after stimulation and thus reveals tip localization, a value = 1 indicates no difference of overlap of Slp2-a with VWF after stimulation and a value > 1 indicates more overlap of Slp2-a with VWF after stimulation. N = 21 cells of 3 different experiments; error bars = SD. Significance was tested with unpaired student's t-test (** $p \leq 0.01$).

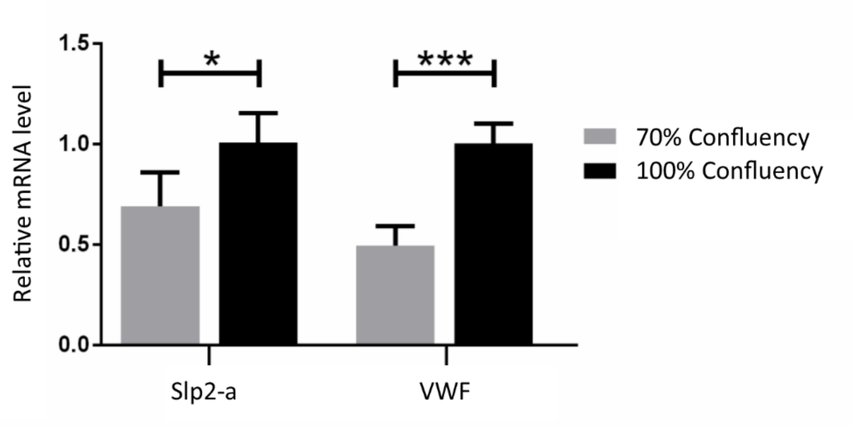

Figure S6. Confluency effects Slp2-a mRNA level.

HUVEC were grown either to 70% or 100% (full) confluency. mRNA was isolated and analyzed via quantitative RT-PCR. β-actin and β-2-microglobulin were included as housekeeping genes. n = 4 experiments, significance tested with unpaired Mann-Whitney test (Slp2-a) or student's t-test (VWF) (*$p \leq 0.05$; ***$p \leq 0.001$).

Video 1 HUVEC transfected with VWF-GFP were imaged with simultaneous TIRF (red) and epifluorescence (epi, green) illumination, and stimulated with 20 μM histamine during acquisition. Video shows lateral fusion of WPB. Frame rate: 50 frames per second.

Video 2 HUVEC transfected with VWF-GFP were imaged with simultaneous TIRF (red) and epifluorescence (epi, green) illumination, and stimulated with 20 μM histamine during acquisition. Video shows tip fusion of WPB. Frame rate: 3 frames per second.

Video 3 HUVEC transfected with Slp2a-wt-GFP (green) and VWF-RFP (red) were imaged by confocal time-lapse microscopy and stimulated with 100 μM histamine during acquisition. Frame rate: 12 frames per second.

Video 4 HUVEC transfected with Slp2a-ΔC2AB-GFP (green) and VWF-RFP (red) were imaged by confocal time-lapse microscopy and stimulated with 100 μM histamine during acquisition. Frame rate: 12 frames per second.

Video 5 HUVEC transfected with Slp2a-wt-GFP (green) and VWF-RFP (red) were imaged by confocal time-lapse microscopy and stimulated with 400 μM histamine during acquisition. The Slp2-a-positive tip of WPB is the site of fusion initiation. Frame rate: 12 frames per second.

Video 6 HUVEC transfected with Slp2a-DN-GFP (green) and VWF-RFP (red) were imaged by confocal time-lapse microscopy and stimulated with 500 μM histamine during acquisition. Frame rate: 5 frames per second.

Video 7 HUVEC transfected with Slp2a-KQ-GFP (green) and VWF-RFP (red) were imaged by confocal time-lapse microscopy and stimulated with 500 μM histamine during acquisition. Frame rate: 5 frames per second.
